# Lecture on Clinical Medicine, Delivered at the Philadelphia Medical Institute

**Published:** 1838-08-15

**Authors:** W. W. Gerhard

**Affiliations:** Physician to the Philadelphia Hospital, &c.


### CLINICAL LECTURE.

A LECTURE ON CLINICAL MEDICINE, *delivered at the Philadelphia Medical Institute, by* W. W. Gerhard, M. D., *Physician to the Philadelphia Hospital*, &c.

### LARYNGITIS.

Continuing the subject of diseases of mucous membranes, I shall, to-day, consider some of the affections of an organ, the healthy action of which is most important to life,—I mean the larynx. Laryngitis may occur as an acute disease, the symptoms of which are exceedingly simple, and I shall make but few remarks upon it. It occurs both in adults and children, and is rarely fatal; its pathological characters consist in inflammation of the mucous membrane, which is injected, and thickened, to a sufficient extent, to impede respiration. The act of respiration is, in consequence, accompanied by a peculiar stridulous sound, and the tones of the voice altered, in a decided manner. Another symptom of the affection is pain, which, you know, is always present in severe mucous inflammations, although less acute and less limited than in those of the serous membranes. The membrane of the larynx is irritated by the passage of the air over it, which occasions a dull pain, of more or less severity, but not lancinating. This pain is increased by deglutition, from the action of the muscles of the pharynx.

The condition of the voice is an important point in the diagnosis; it is stridulous, which is never the case in simple inflammation of the lungs; shrillness is peculiarly characteristic of laryngeal inflammation; as the disease goes on, the voice is either lost, or resumes its tones by the recovery of the patient.

Laryngitis may be confounded with simple inflammation of the tonsils, from which it may be distinguished by the absence of the nasal sound of the voice, which, in tonsilitis, is caused by the air being cut off, at the posterior part of the pharynx, in its passage through the nose. The diagnosis of laryngitis is a very easy affair, and I shall not detain you with it any longer.

The treatment proper for acute laryngitis, is laid down by every author who has treated of the subject, and I shall not take up your time, by detailing the various antiphlogistic remedies which may be resorted to; laryngotomy, I may remark, is rarely necessary.

The croupal variety of laryngitis, or that in which a false membrane is thrown out, may be subdivided into two kinds. In the first, the formation of the false membrane commences in the pharynx, and this variety is to be recognised by examination of the throat, before the larynx becomes implicated; if it extend down into the latter, unchecked by art, the affection is mortal. In this type of laryngitis, the voice is more shrill than in ordinary cases, or the patient is aphonous. In the treatment of this variety, antiphlogistics are to be resorted to, though they are not alone to be depended on. Local applications are to be made to the pharynx, such as a strong solution of nitrate of silver, or of alum, or the muriatic acid; a sponge may be dipped in these liquids, and applied to the part, which is to be afterwards gargled with flax-seed tea. It behoves us entirely to destroy the false membrane and prevent its extending down into the windpipe, where a slight obstruction is fatal.

The last variety of acute laryngitis is the croupal, in which the secretion of the false membrane commences below, and not above, as in diphtheritis. It is rarely confined to the trachea, but extends much further into the bronchial tubes of both lungs; in this variety, the voice is stridulous and shrill, and, in young children, there is a peculiar cry; there is also cough. The respiratory sound in the chest is feeble, from the difficulty offered to the free entrance of air into the lungs; there are other signs of the affection, such as a flushed, swollen face, and dyspnœa, with incessant restlessness and jactitation. In this variety of laryngitis, so much dreaded in children, the rules of treatment are so well laid down, and variety of opinion is so limited, that I shall not detain you by pointing them out at length. It is not to be treated as diphtheritis, or spasmodic croup. Bleedings, general and local, nauseating remedies, as tobacco externally applied, are beneficial in both varieties; but the croupal is an exceedingly difficult one to cure.

Subacute laryngitis has lately occupied much of my attention. It usually occurs in subjects afflicted with previous disease of the lungs; and, when it assumes this secondary shape, it is very apt to be mortal, if suffered to advance, for no patient can resist so extended an interruption of respiration. In these cases, it is the duty of the physician to interfere with promptness. An active depletory treatment is, however, scarcely called for; but we rely upon external irritants over the larynx. Emollient fumigations are useful; and when the disease arises in the pharynx, and passes downwards towards the larynx, as in diphtheritis, it is always proper to apply a stimulant wash to the pharynx. Tracheotomy may be resorted to, after the caustic has been ineffectually applied. Tracheotomy is a remedy of doubtful propriety; the use of it has been abandoned in genuine croup; but it is resorted to in diphtheritis, where the inflammation has not yet extended from above downwards to the bronchi, and there are indications of a sound condition of the lower part of the windpipe.

Chronic laryngitis is the most common form of the affection, and I propose to dwell upon it at greater length than upon the other varieties. This disease has been sometimes termed laryngeal phthisis; but I shall be disposed to doubt the propriety of universally applying this term, since the laryngitis often occurs before the developement of phthisis pulmonalis, as was the case in a patient who has just left the hospital. Indeed, it may sometimes continue for a long period, without any signs of pulmonary disease. Now, in these cases, the original point of disease is in the larynx, although the lungs rarely escape, especially if the affection be much protracted, or if the patient be of an original tuberculous constitution.

The patient, whose history I shall make the subject of this lecture, has been very dissipated in his habits, having twice had syphilis, and been salivated severely for it each time.

The patient entered the hospital, June 4th, 1838. He is an Irishman, a single man, originally a sailor, but broke his leg, and, for the last three years, has been a shoemaker. Has drunk freely, from boyhood, particularly for the last two years, but has never had delirium tremens. Has had the venereal four times; has been twice salivated, the last time, about the middle of last November—recovered entirely, and never before had sore throat or secondary symptoms. Never had a *cold* before, nor ever sick; has always been a stout, healthy fellow, excepting during a temporary illness, in a tropical climate. Began to cough in September, 1837, having been previously quite well, but, after drinking hard, slept out in the grass, for several nights. Had no pain; but, nearly at the same time, had cough and hoarseness. The cough has been, on the whole, increasing, so has the hoarseness; and, since Christmas, the voice, which is naturally clear, and, during the whole autumn, has been rough, has been almost entirely lost. Soreness of the throat began about four days after the cough, opposite the depression beneath the hyoid bone. No pain at the sternum, nor any in the chest, except six weeks ago, when it was slight at the anterior part of the right axilla, and was speedily removed by a blister.

The patient went to the Camden races in October, drank very freely, and contracted gonorrhœa and chancres; was slightly salivated; became more hoarse, and coughed more. Went to work at his trade, until the 22d of March; did nothing for his cough, until he recovered from the venereal, a month after the infection, when he took simple remedies, as honey, &c. On the 22d of March, he entered the Pennsylvania Hospital, where he remained until two days before he came to our hospital; during that time, he had less soreness of the throat, and less tumefaction of the glands. The cough continued, with greater severity.

Expectoration began, before he went to the races; he spit about half a teaspoonful of blood, clotted, appearing to come from the throat. Appetite good, until about the first of March, and he drank less spirits. Bowels *never loose*; regular. No pain in the belly, nor uneasiness, after eating. No night sweats. Emaciation began immediately after the venereal. No sore legs. Never had epistaxis, except twice, in last winter.

At the Pennsylvania Hospital, he was leeched twice to the throat, blistered constantly for two weeks, and took some opiate mixture. Swallowed with difficulty at first, but now more readily.

At the time of his admission, his condition was as follows. Emaciation. Light chestnut complexion. Height five feet seven inches. No swelling of the limbs. Strength feeble; can readily walk up stairs, and thinks he can walk half a mile. Much dyspnœa. Pulse 100, feeble, regular; hands cool. Tongue moist; slightly coated. Appetite good. Abdomen retracted; bowels regular; one or two stools daily. Aphonia nearly complete. No pain in the throat, but some tenderness. Uvula thickened, enlarged; slight redness of the fauces only. Cough stridulous; frequent. Expectorates eight or ten ounces of puriform and nearly numular matter. He was ordered lime water and milk, the infusion of the prunus virginiana, and inhalations of laudanum and water.

The physical signs, afforded by an examination, the day after the patient's admission, were the following:—Posteriorly, there was flatness on percussion at the summit of the left side; dulness at the middle, but below it was tolerably clear. On the right side, it was clear, in the lower half, but became flat towards the summit. The respiration on the left side was cavernous at the summit, bronchial at the root of the lung, for the extent of two inches; feeble, but vesicular, inferiorly. Pectoriloquy at the summit, notwithstanding the alteration of the voice. On the right side, cavernous respiration at the summit, rude at the root, vesicular at the lower half of the lung. Anteriorly, there was flatness at the summit, on both sides; contraction between the clavicles. On the left side, some vesicular sounds were still heard at the clavicle, with distinct cavernous respiration; a little crackling. On the right side, the respiration at the clavicle was cavernous, without vesicular sound, and without crackling.

I shall not occupy your attention with the details of the progress of this case, but will merely mention the symptoms at the time of the man's discharge, the 27th of June. The intermediate treatment, in addition to that mentioned, consisted in occasional doses of blue pill, with some acetate of lead and opium, for a diarrhœa which came on, and which was arrested. At the time the man left the hospital, his aphonia was rather less; he was feeble, and had irregular sweats. Bowels regular. Cavernous respiration very pure, under the right clavicle; at the left, crackling, with rude respiration; loud and puerile, at the lower portion of the left lung; feeble with mucous rhonchus, at the lower part of the right. Discharged, at his own request.

I shall not particularly dwell upon the case, the history of which I have just given you, but, instancing it merely as an example of the affection under notice, I shall enter into some general remarks on the subject.

Chronic laryngitis is associated with pulmonary phthisis in several ways. The affection of the larynx may be strictly secondary, and result from the direct irritation of the sputa passing over the bronchial tubes and the mucous membranes of the larynx; this form of the disease occurs in the more advanced stages of phthisis when the sputa are more irritating than at the commencement, before softening of the tuberculous substance has occurred. In other cases, the tuberculous disease appears in the larynx nearly, if not quite as early, as in the lungs, and arises from a deposit of tuberculous matter in the follicles of the larynx. This variety is strictly analogous to the tuberculous affections of the serous or mucous membranes which so often accompany phthisis; that is, the tuberculous disease of these various organs is in reality but a part of one and the same affection, which merely shows itself in many points nearly at the same time. The third variety is the most interesting, and is the one offered by the case which I have just detailed to you. The affection of the larynx does not here seem to be originally of a tuberculous character, but may be either a simple chronic inflammation or one dependent upon a syphilitic taint. The pulmonary affection is of later date, and only appears as a consequence of the irritation which is developed in a continuous mucous membrane. Not that all cases of chronic laryngitis necessarily terminate in this way; it is only those which occur in individuals who are disposed to phthisis, either from original constitution or acquired habits.

Phthisis may not follow this variety of chronic laryngitis for a very long period after its developement. In the case which forms the illustration of this lecture, although there can be no doubt as to the order of time in which the affection of the larynx and lungs appeared, there is a less distinct interval than in many patients. I have seen a number of cases in which the lungs were undoubtedly free from any appreciable signs of disease for months after the laryngeal affection had taken place. In all probability, the lungs were as free from actual as they were from all appreciable lesions, for the general symptoms of phthisis did not appear until about the period when the local signs were discoverable.

I know of no certain rule by which you can distinguish those cases of chronic laryngitis which are to pass into phthisis, from the more simple forms of the disease, in which the ulceration of the mucous membrane and the caries of the cartilages are the only lesions. You should therefore recollect, when you meet with a case in which a careful exploration of the chest convinces you that the patient has no pulmonary affection, how very apt true phthisis is to follow chronic disease of the larynx, and you should not give the patient to understand that his lungs are entirely out of danger. In fact, such cases are often but the prelude or the first stage of a variety of phthisis, the symptoms of which persist until the death of the patient. From a neglect of this necessary caution, or rather, from not being fully impressed with the connection which exists between the early and the more decided stages of the irregular varieties of pulmonary phthesis, I have known physicians of high standing commit grave errors, which proved injurious to the health of their patients, as well as to their own reputation. These errors are sometimes committed by physicians who are familiar with the physical means of exploration, and who have acquired that power of diagnosis which is possessed only by those who are thoroughly conversant with the pathology of pulmonary disease; you may readily imagine how much more frequent they must be with practitioners who diagnosticate disease merely from a few of the most obvious symptoms.

There is no difficulty in the diagnosis of chronic laryngitis; but there is much difficulty in distinguishing its different varieties. For instance, if you meet with a case in which there has been pain and difficulty of deglutition felt at the region of the larynx, and an alteration of the voice which may be limited to a mere huskiness scarcely observable by any one not previously acquainted with the natural voice of the patient, you should regard the case as one of chronic laryngitis, if it persists longer than a week or two. The absence of any obvious alteration of the pharynx does not prove that the larynx is in a normal state; although, when the pharynx is diseased, the larynx rarely escapes for a long period without participating in the evil, at least to some extent. The anatomical changes, occurring in this early stage, are limited to a mere thickening of the mucous membrane, especially that covering the vocal chords; it is sometimes so slight as to disappear entirely after death. This stage of the disease is that which is most frequently followed by phthisis, or, to be more strictly logical, we should perhaps say, that, at this stage, phthisis generally supervenes.

You will find the diagnosis of the following stages still more easy than at the period of ulceration. The ulcers are most common near the vocal chords, in the variety which attends phthisis; but in syphilitic laryngitis, the epiglottis is commonly attacked and very often the cartilages of the larynx become necrosed. By depressing the base of the tongue very firmly, you can often obtain a view of the tip of the epiglottis, and thus ascertain if it present the white or greyish ulcer of syphilis. Still in the laryngitis accompanying phthisis, the epiglottis is not unfrequently ulcerated, so that this sign is not infallible. The peculiar grayish colour of the ulcerations of syphilis, and the simultaneous occurrence of ulcers on the pharynx and tonsils of a similar aspect, are better guides. But we are, after all these precautions, obliged to trust largely to the commensurative circumstances, especially to the signs of syphilitic or tuberculous disease in other organs. There will then remain a few cases of doubt; for instance, the patient whose case is mentioned to-day, has, undoubtedly, had syphilis more than once, and is, evidently, now labouring under a confined phthisis. In his case, I regard the laryngitis as generally of a syphilitic character, but the phthisis very speedily added a new source of irritation to the larynx.

Besides these cases of chronic laryngitis, there are others in which it is very difficult to distinguish how much of the symptoms is owing to the affection of the larynx, and how much to that of the pharynx and trachea which may accompany it. These cases constitute the annoying disease which has been sometimes called clergymen's sore throat. This designation it has received from its frequent occurrence in the members of the clerical profession. It is now rather less frequent than formerly, and is passing from out of the list of fashionable complaints, so that in a few years we shall probably scarcely hear of its appearance. In speaking of it as a fashionable disorder, I do not mean to jest about a very annoying complaint, one that is quite inexplicable, and which indirectly leads to serious consequences.

This variety of sore throat which occurs so often in clergymen, is by no means very rare in other professional men who are engaged in pursuits little calculated to promote vigorous health, and are at the same time obliged to exert their voice in addressing large audiences. There is no doubt that a feeble constitution, especially if inclined to scrophulous disorders, favours in a remarkable degree the sore throat, but, notwithstanding, I have often seen men of a vigorous, and apparently altogether firm constitution, suffer extremely from this affection. The liability of clergymen to this kind of sore throat depends upon several causes; in the first place, the duties of a clergyman in this country are sometimes unreasonably arduous. In many cases his parishioners expect that, in addition to the ordinary duties of his calling, he should take an active part in many religious societies and meetings, which make large demands upon the time that should be devoted to active exercise and cheerful recreation. The origin of the feeble constitution of clergymen in many cases depends upon still more remote causes, and arises from an absurd neglect of the ordinary rules of hygiene while pursuing their preparatory studies. When you reflect upon the influence of these sources of disease, and add to them the feeble constitution that not a few clergymen possess from original habit of body, you may understand the reason why the clergymen in our cities enjoy less perfect health than any other class of professional men. In Europe it is well known that the reverse is universally true.

The affection of which I am now speaking rarely offers laryngitis in its acute form. Indeed it generally commences in the pharynx, and extends in a secondary manner to the windpipe. Its exciting cause may be either an attack of acute fever, especially scarlatina, or it may occur as an ordinary angina which is prolonged beyond the usual duration of these affections. When we examine the pharynx we find it to be red, smooth, and irregularly elevated; the redness of the membrane extends also into the larynx, and may be detected at the tip of the epiglottis. When these cases are either acute or have lasted for a long period, the trachea and larynx will be found painful on pressure, and the voice becomes feeble and hoarse.

The lungs often become tuberculous after the irritation of the pharynx has lasted for some weeks, but you must not suppose that there is any necessary connection between phthisis and this affection; it is a more exciting cause, but not a frequent precursor of the disease, as is the case with true chronic laryngitis. It is quite surprising how long these cases will last without the lungs materially suffering, and unless the general and local signs of phthisis are both evident, you must not conclude that your patient is in the early stages of consumption.

(To be continued.)

